# Possible effects of melatonin and omega-3 on the obesity-related hypothalamic nuclei of the electromagnetic field-exposed offspring rats: a stereological and immunohistochemical analysis

**DOI:** 10.3389/fpubh.2025.1583097

**Published:** 2025-06-25

**Authors:** Gamze Altun, Süleyman Kaplan

**Affiliations:** Department of Histology and Embryology, Faculty of Medicine, Ondokuz Mayıs University, Samsun, Türkiye

**Keywords:** electromagnetic field, childhood obesity, hypothalamic nuclei, melatonin, omega-3, optical fractionator

## Abstract

**Aim:**

The main aim of the study is to qualitatively and quantitatively evaluate the neuronal changes that occur in the hypothalamic nuclei of newborn male rats related to obesity during the intrauterine period, which was exposed to electromagnetic field (EMF). It was also investigating the expressions of obesity-related neuropeptide Y (NPY) and fat mass and obesity-associated gene (FTO) in the arcuate (ARN), ventromedial (VMN), and dorsomedial nuclei (DMN). It was also aimed to examine the role of omega-3 (ω3) and melatonin (Mel) against the side effects of EMF.

**Methods:**

Adult Wistar albino pregnant female rats were randomly divided into seven groups: Cont, Sham, EMF, EMF-Mel, EMF-ω3, Mel, and ω3. While no treatment was applied to the control group, rats in the Sham group were kept in the cage system for 2 h per day for 21 days without exposure to EMF. EMF groups were exposed to 900 MHz EMF for 2 h per day during pregnancy. Mel-treated groups received 50 mg/kg/day melatonin, while ω3-treated animals were given 0.93 g/mL ω3 via intragastric gavage. Anxiety and locomotor behaviors were assessed. Oxidative stress parameters were analyzed. Stereological, immunohistochemical, and ultrastructural analyses were performed on the offspring rats’ hypothalamus.

**Results:**

It was seen that serum superoxide dismutase activity was significantly higher in the ω3 group compared to the other groups (*p* ≤ 0.01), and serum catalase activity was significantly higher in the EMF group compared to the Cont and EMF-ω3 groups (*p* ≤ 0.01). The number of neurons in the ARN was significantly lower in the EMF group compared to the Sham group (*p* ≤ 0.05). According to the open field test, the time spent in the peripheral zone in the EMF group was longer than in the Cont group (*p* ≤ 0.05); In the elevated plus maze test, the number of entries into the open area in the EMF group was lower than in the Cont group (*p* ≤ 0.01). Stronger anti-NPY immunoreactivity was observed in the EMF group.

**Conclusion:**

Prenatal exposure to mobile phones may have hypothalamic effects by inducing neurodegeneration and affecting FTO and NPY expressions. The possible therapeutic effects of Mel and ω3 were not apparent.

## 1 Introduction

The discovery of new communication technologies, such as mobile phones, has brought significant momentum to our daily lives. In addition, facilities established due to the developing telecommunications industry may lead to various health risks (1, 2). The use of 5G technology raises concerns about its effects on human health. The widespread use of wireless devices, a significant source of radiofrequency electromagnetic fields (RF-EMF), can lead to behavioral problems, especially in children and adolescents (3). Since the lifetime exposure period is more extended in children and adolescents than in adults, it has been suggested that children are prone to environmental exposure due to their developing nervous systems. In addition to this source increase, the adverse effects of exposure to electromagnetic fields (EMF) are a concern due to the widespread use of mobile phones at an early age. The possible effects of EMF on the developing nervous system have been investigated (4, 5). Evidence on the effects of EMFs emitted from sources such as microwave ovens and mobile phones on biological systems suggests that mobile phone use may also have health effects. EMF exposure is considered under two headings: low-frequency EMFs emitted from electrical and electronic devices, and radio frequency (RF) (800–2000 MHz) radiation emitted from mobile phones and cordless phones. Low-frequency EMFs have low frequency power; however, when the magnetic flux density is over 0.2 or 0.3 μT (6, 7). In addition, both EMF types are considered non-ionizing radiation (7). Epidemiological and experimental studies are noteworthy when considering the effect of EMF emitted by mobile phones on brain functions, mainly due to their use close to the brain (8–12). Acute exposure to 900 MHz EMF signals can cause molecular and cellular changes in brain cells (13).

The cognitive effects of EMF exposure in adolescence and childhood are known (11, 14, 15). It is suggested that behavioral activities increase, but memory is weakened in newborn mice exposed to RF-EMF during the prenatal period. There are also evaluations that EMF exposure may lead to hyperactivity and memory impairment (16). In addition, different studies have shown that EMF can cause many changes in brain energy metabolism and the metabolism of reactive oxygen species (ROS) (17–20). In addition to functional and morphological findings, it has been determined that EMF also affects metabolic processes in the brain (21). In the case of long-term exposure to EMF emitted from mobile phones, an increase in glucose metabolism was found in positron emission tomography scanning (21, 22). EMF exposure on glucose metabolism and endocrine effects during puberty has been discussed (23, 24). The response of different cells to stress caused by EMF under various conditions and the underlying molecular mechanisms enable the effects of EMF on disease risk to be measured (7).

Previously, the widespread use of mobile phones in society coincided with the increase in human body weight and obesity worldwide (18, 25, 26). In recent years, Wardzinski et al. (18) suggested that RF-EMF exposure triggers carbohydrate intake in humans, reporting increased cerebral high-energy phosphate metabolism after exposure. RF-EMF emitted from cell phones is mainly absorbed in the head region. Increased brain glucose metabolism has been observed with mobile phone exposure (21). Glucose-sensing neurons in the hypothalamus are critical in general energy homeostasis and feeding behavior (27). Impairments were detected in the glucose-sensing neurons of rats exposed to EMF and fructose intake, and therefore, it was suggested that there was a loss in satiety control. Fructose intake combined with exposure to RF-EMF (daily 2 h) during childhood–adolescence affects the central nervous system regions responsible for feeding behavior in adulthood (28). Similarly, Bektas et al. (29) reported that RF-EMF led to increased ghrelin and decreased nesfatin-1 levels in the brain. In this regard, they suggested that 3.5 GHz RF radiation caused changes in appetite and energy homeostasis of diabetic and healthy rats (29).

Since the developing brain is more sensitive to toxins and can absorb more RF energy, the risk is higher in juvenile rats than in adult rats. In this context, the current study aimed to examine the effects of EMF exposure in utero on the developing brain. EMF emitted from mobile phones may cause significant changes in the hypothalamus regarding neurodegenerative effects and protein expressions (30, 31). In addition, when the potential oxidative effects of RF-EMF are examined, the effects may differ depending on the frequency, amplitude, waveform, and changes in polarization of the EMF (32–34). RF-EMF can cause significant disruption in mitochondrial functions. This disruption can be observed due to increased ROS production after exposure to RF-EMF (35–37). It is known that the disorder in mitochondrial oxidative metabolism leads to neuronal dysfunction and associated neurodegeneration (38). In EMF exposure studies conducted with cellular and experimental animal models, it has been found that autophagy and mitochondrial oxidative mechanisms are activated (39).

Genetic factors contribute to the risk of developing obesity. One of them is FTO (fat mass and obesity-associated gene) and is mainly expressed in the hypothalamus, which is known as the regulatory center in appetite balance (40, 41). Therefore, it is essential in circadian rhythm, anxiety-like behaviors, energy homeostasis, and memory function. Neuropeptide Y (NPY) levels, a neuropeptide commonly found in the brain, have been observed to be reduced in conditions such as depression, chronic stress, and anxiety (42). Previously, high concentrations of obesity-related NPY levels in the hypothalamus were found in the male rats exposed to melatonin (Mel) during the prenatal period, up to 40 days of age (43). Considering the anti-obesity property of Mel, it has been suggested in recent years that it reduces the orexigenic effect by reducing NPY/Agouti-related peptide (AgRP) in the hypothalamus (44). Similarly, omega-3 (ω3) fatty acids led to an increase in orexigenic NPY expression (45).

The effects of EMF on hypothalamic FTO and NPY expression in offspring rats after prenatal exposure may provide new evidence on the predisposition to obesity. In addition, since NPY is closely related to depression and anxiety, it may lead to conclusions that EMF emitted from mobile phones triggers anxiety in infant rats. The results obtained from the interaction of Mel and ω3 with EMF in the infant rat brain, which provide data on their neuroprotective and anti-obesity effects, will provide new information on these two therapeutic agents. However, there is not enough information on the effects of Mel and ω3 on FTO expression. The results obtained from the interaction of Mel and ω3 with EMF exposure in infant rat brains, which provide data on their neuroprotective and anti-obesity effects, will reveal new information about these two therapeutic agents. Therefore, comparing the results on the interaction of Mel and ω3 with EMF exposure may provide critical information for developing treatment options. Acute or chronic exposure to 900 MHz EMF can affect brain activity regionally (13, 46). In the design of the experimental exposure model, the frequency of 900 MHz, a band widely used in mobile telephone systems, was chosen (47). Since the 915 MHz system is less energy efficient, it requires longer exposure times than, for example, the 2.4 GHz system (48).

In this study, male offspring rats were preferred because it was anticipated that hormonal changes resulting from the menstrual cycle of female rats could affect the analysis results. There are limited studies on the neuroprotective or neurogenesis-stimulating effects of Mel and ω3 against hypothalamic apoptosis and obesity development in the central nervous system. This situation requires more comprehensive experimental studies to explain the possible roles of Mel and ω3 on hypothalamic neurogenesis and the underlying mechanisms. The presented study is designed to determine whether Mel and ω3 application are protective against possible neurodegenerative effects caused by 900 MHz EMF. This study investigated the effects of 900 MHz EMF exposure in the prenatal period on the hypothalamic nuclei and hippocampus using stereological, histopathological, immunohistochemical, biochemical, and behavioral methods. Possible degenerative changes that may be caused by exposure to EMF during the prenatal period and the tendency to obesity that may occur in the postnatal period in the offspring rats were investigated.

## 2 Materials and methods

### 2.1 Animals and ethical approval

All experimental procedures conducted in this study were approved by the ethics committee decision of the Local Ethics Committee for Animal Experiments of Ondokuz Mayıs University, dated 05.04.2018 and numbered 2018/24. Power analysis was performed using Minitab (version 18.0, UK) to determine the appropriate number of animals for the groups. Following the ethics committee approval, thirty-five 10–12-week-old adult female Wistar albino rats were obtained from the Experimental Animals Application and Research Center. Adult female rats were randomly divided into seven groups, with five animals in each group. Then, these animals were allowed to mate with male rats in separate cages under appropriate conditions for one day, and the vaginal smear sample taken from the female rats was examined the next day. For the rats where sperm was detected in their samples, it was accepted as day zero of pregnancy. After the pregnancy diagnosis, rats were divided into groups and placed in separate cages. Pregnant rats were kept in a room with a humidity of 40–50% and a room temperature. The rats were kept in an environment with a 12-h dark/light cycle. During the experiment, animals had free access to standard pellet feed and water (tap water).

**Figure 1:**
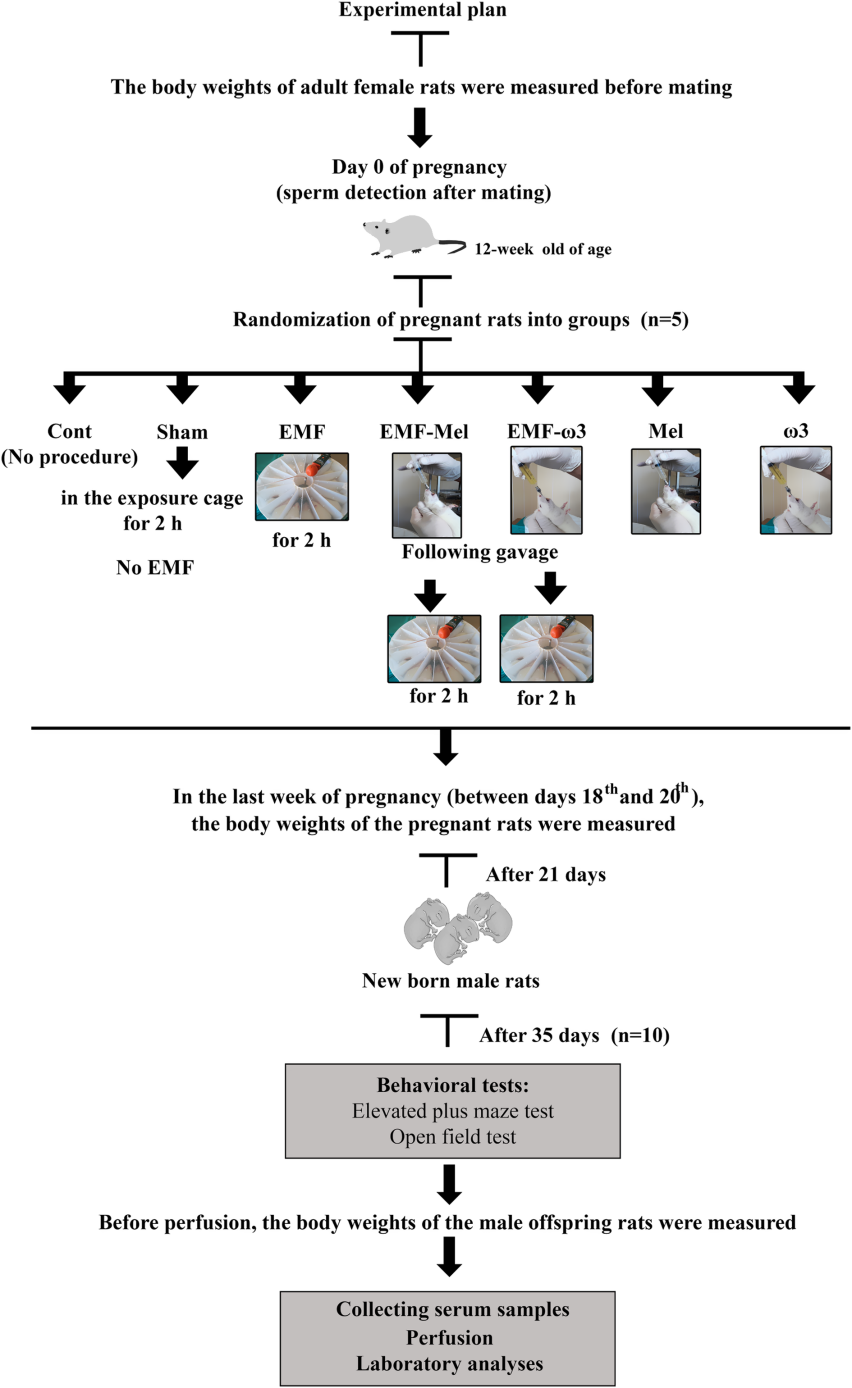
Schematic illustration of experimental design.

Animal groups consisting of adult pregnant rats that are the Cont group, the Sham group, the EMF-exposed group (EMF), the group administered 50 mg/kg/day Mel by intragastric gavage before EMF exposure (EMF-Mel), the group administered 0.93 g/mL ω3 (F8020, Sigma-Aldrich, United States) by intragastric gavage before EMF exposure (EMF-ω3), the group not exposed to EMF but administered 50 mg/kg/day Mel (Lyophilized Melatonin Powder M5250 10 g, Sigma-Aldrich, United States) (Mel) and the group not exposed to EMF but administered 0.93 g/mL ω3 (ω3). Preparation of a 50 mg/kg/day Mel (Lyophilized Melatonin Powder M5250 10 gr, Sigma Aldrich, United States) solution was done by considering the body weights of the rats. A 12.5 mg powdered Mel was weighed per animal in rats in the EMF-Mel and Mel groups. The solution was prepared fresh for 10 pregnant rats daily and given via intragastric gavage. However, 1 mL of ω3 (Sigma Aldrich, United States) prepared from a concentration of 0.93 g/mL was administered to each pregnant rat in the EMF-ω3 and ω3 groups via intragastric gavage. Fifteen pregnant rats were exposed to 900 EMF (Figure 1).

In this study, EMF exposure was created in the EMF group, and stress exposure was created in the Sham group, and antioxidants such as omega-3 and Mel were applied to the mothers of the offspring rats. The effects of such exposure were examined in the male offspring rats (35-day-old). No antioxidant or EMF application was applied to male offspring rats in the postnatal life. At the end of the gestation period, the mothers gave birth, and the offspring rats stayed with their mothers for 21 days after birth. Newborn male rats were not exposed to any treatment during postnatal life. A total of 70 rats were tested. At the end of this period, the animals were sacrificed.

Then, in the last week of pregnancy (between the 18th and 20th days), just before birth, the body weights of rats belonging to the Cont, Sham, EMF, EMF-Mel, EMF-ω3, Mel, and ω3 groups were measured again.

### 2.2 EMF exposure setup

**Figure 2:**
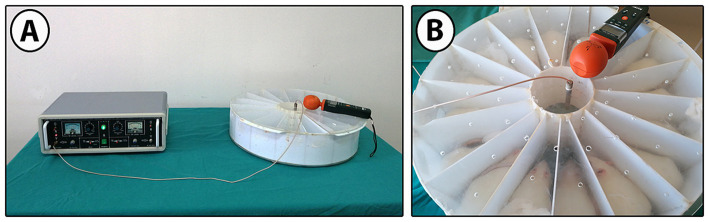
**(A)** 900 MHz EMF signal generator, EMF exposure system, and **(B)** EMF exposure cage with 16 equal compartments.

After the groups were formed, pregnant mother rats were exposed to 900 MHz simultaneously every day for two hours a day for 21 days. EMF exposure was measured as signal strength: 40.5053 V/m, power density: 4.3519 W/m^2^. As an EMF source, an EMF exposure system with a 0–9 W output signal generator emitting 900 MHz RF waves was used (49–51) (Figure 2). The monopole antenna used to collect RF waves from a single source was placed in the middle of the 16-section Plexiglas experimental setup, 20 cm high and 25 cm in radius. EMF, EMF-Mel, and EMF-ω3 rats were exposed to 900 MHz EMF in the experimental setup. Before the EMF generator was started, the electric field value of the environment was measured. During the experiment, 20 measurements were recorded every six minutes during the two-hour EMF exposure. The distance of the cephalic region of the rats to the antenna was measured as 3.6 cm on average, the distance of the abdominal region to the antenna was 7 cm on average, and the distance of the caudal region to the antenna was 9 cm on average. An EMF meter (RF-EMF strength meter, Extech Instruments Corporation, United States) was used to measure the electric field value at the closest point from the antenna to the target tissues in the cephalic, abdominal, and caudal regions.

### 2.3 Behavioral tests

In this study, to investigate the emotional and locomotor effects of EMF exposure, the neuroprotective activity of Mel and ω3, as well as their possible effects on emotional and locomotor activity, 35-day-old male offspring rats were subjected to the open field test and elevated plus maze test. Behavioral tests were performed using appropriate apparatus at the Ondokuz Mayıs University Experimental Animal Application and Research Center, and the behaviors of the animals in all groups were recorded with a camera. The open field test is applied to evaluate the emotional state of male offspring rats, anxiety-like behaviors, and locomotor activity (52, 53). In the test, when rats are left in an open area for five minutes, being placed alone in a different environment triggers anxiety. Defecation and scratching behaviors within five minutes are considered when analyzing vertical and horizontal movements.

Horizontal movements refer to the rat’s transition from one frame to another; movements observed in the vertical plane are considered as the rat rising on its hind limbs (54). The study calculated the time spent by young male rats in the periphery (outside area) and center (inside area), defecation, itching numbers, and movements in horizontal and vertical planes. The test setup consists of 49 equal squares with dimensions of 100 cm x 100 cm x 30 cm. The elevated plus maze test setup, one of the tests used to examine emotional activity, is 50 cm high and consists of two open and two closed arms. Before the test, the subjects were allowed to explore the setup for five minutes. The number of times they enter open areas and the total time spent there were considered when measuring the anxiety index. High anxiety is expressed with a low index. In addition, the time spent moving from the closed area to the open area and the short time spent in the open area indicate the presence of anxiety (55).

### 2.4 Perfusion and serum collection

The body weights of male offspring rats were measured and recorded before perfusion. Thirty-five days after birth, male offspring rats were anesthetized for intracardiac perfusion by intraperitoneal (i.p.) injection of a ketamine (50 mg/kg)/xylazine (10 mg/kg) mixture prepared in a 5/1 ratio. Before perfusion, 1 mL blood samples taken from the hearts of each rat were centrifuged for 15 min at 2000 rpm at +4°C using a centrifuge device (Hermle Labortechnik GmbH, Germany). After centrifugation, the separated serum samples were stored at −20°C for analysis of antioxidant enzyme levels. Brain tissues of the rat that underwent intracardiac perfusion were dissected.

### 2.5 Biochemical analyses

To investigate the antioxidant activity of Mel and ω3 against oxidative stress mechanisms that may occur due to EMF exposure, superoxide dismutase (Cayman Chemical Company, United States) and CAT (Catalase) (Cayman Chemical Company, United States) enzyme activities were analyzed in serum samples taken from the rat at the Department of Medical Biochemistry, Faculty of Medicine, Ondokuz Mayıs University. After the serum samples were incubated in the Nuve SL 350 device for the calculation of enzyme activities, the absorbance values of the solutions reacted in the microplate were recorded on a microplate reader device (Tecan Instruments, Austria) at 450 nm for superoxide dismutase enzyme activity and at 540 nm for CAT enzyme activity.

### 2.6 Paraffin-embedded blocks

After intracardiac perfusion, dissected brain tissues were placed into a 4% formaldehyde (Sigma-Aldrich, United States) solution for fixation. After the fixation process, brain tissues were subjected to a routine tissue tracking procedure. A tissue tracking device was used for routine tracking of brain tissues (Thermon Shandon Citadel 2000, United States). After the tissue embedding procedure, brain tissues were embedded in paraffin in the coronal plane. All tissues were randomly numbered to ensure that all analyses in the study were done blindly.

### 2.7 Stereological analyses

According to the systematic random sampling (SRS) rule, serial sections of 25 μm for stereological analyses and 4 μm for immunohistochemical analyses were taken from the paraffin blocks using a Leica RM2245 microtome (Leica, Nussloch, Germany) at a 1/2 section sampling ratio. Then, the sections placed on slides were kept in an oven at 60°C overnight for deparaffinization of the sections. An average of 30 sections of 25 μm thickness obtained from each block by SRS were stained with cresyl violet. Stereological analyses were performed on these sections. The sections were stained with cresyl violet dye (C5042-10 gr, Sigma-Aldrich, United States). Neuron counting was performed using the optical fractionator method (56) in a computer-aided stereology analysis system with special software (Stereoinvestigator 9.0, MicroBrield Field; Colchester, United States) at the Department of Histology and Embryology, Faculty of Medicine, Recep Tayyip Erdoğan University. The area sampling ratio in neuron counting is 1,600 μm^2^/3600 μm^2^. In addition to neuron counting, the arcuate nucleus (ARN), the ventromedial nucleus (VMN), and the dorsomedial nucleus (DMN) sectional areas were measured using the planimetric method. The sectional areas were multiplied by the section thickness to obtain a volume of hypothalamic nuclei.

### 2.8 Immunohistochemical analyses

After deparaffinization of 4 μm thick sections obtained from each rat brain, immunolabelling of anti-NPY (catalog number: ab30914, dilution: 1:500, Abcam, UK), anti-FTO (catalog number:ab126605, dilution: 1/200, Abcam, UK) and anti-Ki67 (dilution: 1:500, Abcam, UK, catalog number:ab15580) primary antibodies belonging to the Control, Sham, EMF, EMF-Mel, EMF + ω3, Mel and ω3 groups were histopathologically evaluated. A mouse and rabbit-specific HRP/AEC (ABC) detection kit (catalog number: ab93705, Abcam, UK) was used for immunohistochemical analyses. Hematoxylin solution (Mayer’s modified Abcam, UK) was used as a counterstain. A dark, moist environment container was used during the procedures. In immunohistochemical evaluations, section samples taken from each paraffin block were evaluated (*n* = 9). After the sampled sections were stained, an average of five different areas from each section were reviewed, and representative images were used for histopathological evaluation using a microscope (Olympus, BX43, Center Valley, PA, United States) with a camera attachment (Olympus, SC5, Tokyo, Japan).

### 2.9 Tissue processing for electron microscopy and preparing resin-embedded tissue blocks

One brain tissue from each group was fixed in a 4% glutaraldehyde (Merck Millipore, United States) solution for electron microscopic analysis. Hypothalamic regions were dissected with a stereo microscope in a volume of approximately 2 mm^3^ from the brain tissues, and electron microscopic tissue processing was applied. After this process (Araldite CY212, Agar Scientific Ltd., Essex, UK), hypothalamic tissues were embedded in resin and kept in a dark environment overnight. After the centralization process, the tissues were kept in the oven at 45°C- 50°C- 55°C for 30 min and at 62°C for 48 h to ensure polymerization of the resin blocks (57, 58). Brain tissue from one subject (*n* = 1) from each group was embedded in resin blocks to obtain semithin and thin sections for histopathological evaluation.

Semi-thin sections (0.5 μm) were taken from resin-embedded blocks using an ultramicrotome (Thermo Scientific Shandon, United States). The collected sections were stained with 1% toluidine blue and 0.2% sodium borate solution. Then, images were taken from the ARN, VMN, and DMN in the hypothalamus for histopathological examinations using a light microscope (Olympus, BX43, Center Valley, PA, United States) with a camera attachment (Olympus, SC5, Tokyo, Japan) using the computer-aided cellSens Entry microscope software program (Olympus, Center Valley, PA, United States). In addition, 70 nm thin sections were taken with a diamond knife on an ultramicrotome (Leica ultra cut UCT, Leica Microsystems GmbH, Germany) for the examination of the ultrastructure of the cell and were placed on copper grids and contrasted with 0.5% uranyl acetate and 3% lead citrate solution. The obtained micrographs were examined under an electron microscope (JEOL JSM-7001F, JEOL Ltd., Tokyo, Japan) at the Black Sea Advanced Technical Research and Application Center.

### 2.10 Statistical analysis

Statistical analysis of numerical data was performed using GraphPad Prism (version 8.0, GraphPad Software, San Diego, CA, United States). All data were subjected to a normality test using the Shapiro–Wilk test. Normally distributed data were evaluated using One-way ANOVA (Post hoc: Tukey’s test), and non-normally distributed data were evaluated using the Kruskal-Wallis test (Post hoc: Dunn’s test). In data expressed as “mean ± standard deviation,” a significance value of 0.05 (*p*) was considered.

## 3 Results

### 3.1 Body weights

**Figure 3:**
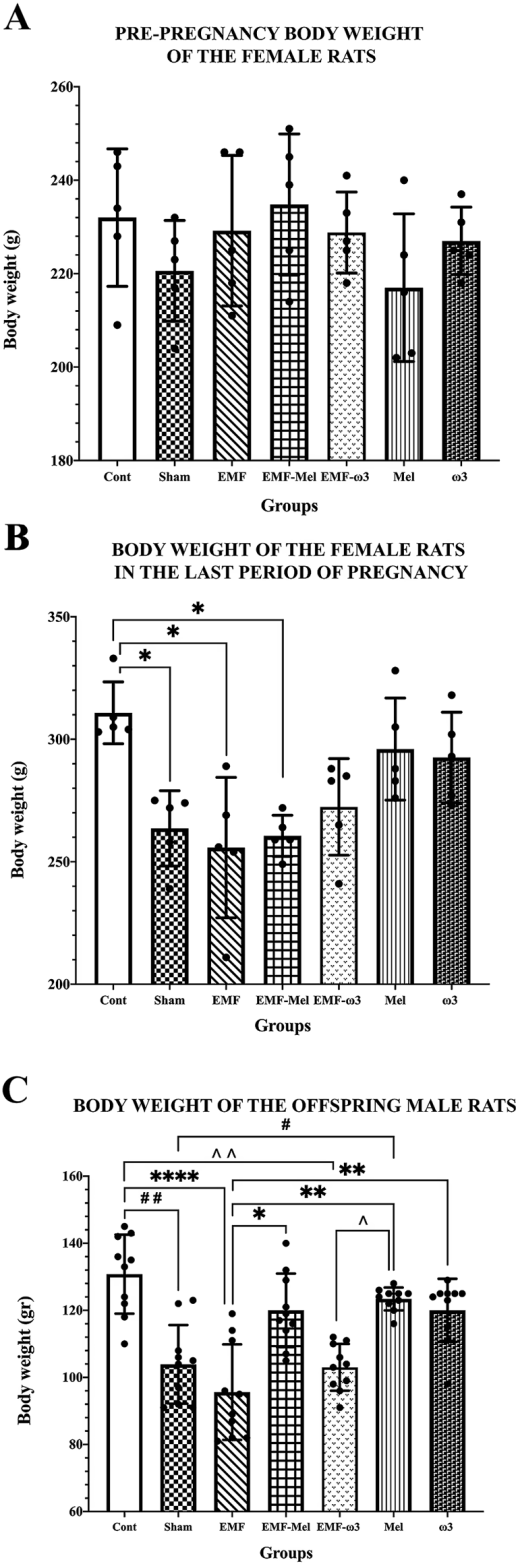
**(A)** Pre-pregnancy body weight values of female rats, **(B)** Body weight values of female rats in the last period of pregnancy, **(C)** Body weight values of 35-day-old male offspring rats are given. **(A–C)** Body weight values of groups are expressed as “mean ± standard deviation.” In the graphs, statistically significant differences at the level of *p* ≤ 0.05 (*, #, ^^), significant differences at the level of *p* ≤ 0.01 (**, ##, ^^), and significant differences at the level of *p* ≤ 0.0001 (****) are shown.

When the pre-pregnancy body weights of pregnant rats were evaluated, no difference was observed between the groups (F (6, 28) =1.143, *p* = 0.3639, One-way ANOVA). However, when the body weights of female rats in the last final period of pregnancy were examined, a decrease was determined in the Sham group compared to the Cont group (*p* = 0.047). However, there was no permanent significant difference between the body weights of female rats in the EMF and the Sham groups in the last pregnancy period (*p* > 0.05). A significant decrease was found when the EMF group was compared with the Cont group (*p* = 0.024). Similarly, a significant decrease was observed in the body weight values of the EMF-Mel group compared to the Cont group (*p* = 0.014). However, there was no significant difference between the EMF-ω3 and the Cont group (*p* > 0.05). It was observed that there was no difference in body weight performance in the ω3 and Mel-treated groups of female rats compared to the Cont group (*p* > 0.05) (Table 1; Figure 3).

**Table 1:** Body weights (g) of the pregnant rats and offspring rats.

Group of animals	Pre-pregnancy body weights of female rats (*n* = 5)	Body weights of female rats in the last period of pregnancy (*n* = 5)	Body weights of male offspring rats (*n* = 10)
Cont	232 ± 14.71	310.8 ± 12.62^*^	130.8 ± 11.76^##, ****, ^^^
Lower 95% CI	213.7	295.1	122.4
Upper 95% CI	250.3	326.5	139.2
Sham	220.6 ± 10.78	263.6 ± 15.37^*^	103.9 ± 11.72^##, #^
Lower 95% CI	207.2	244.5	95.51
Upper 95% CI	234.0	282.7	112.3
EMF	229.2 ± 16.12	255.8 ± 28.67^*^	95.6 ± 14.24^****, **, *^
Lower 95% CI	209.2	220.2	85.41
Upper 95% CI	249.2	291.4	105.8
EMF-Mel	234.8 ± 15.11	260.6 ± 8.39	120 ± 10.96^*^
Lower 95% CI	216.0	250.2	112.2
Upper 95% CI	253.6	271.0	127.8
EMF-ω3	228.8 ± 8.67	272.4 ± 19.72	103 ± 6.98^^^, **, ^^
Lower 95% CI	218	247.9	98.01
Upper 95% CI	239.6	296.9	108.0
Mel	217 ± 15.81	296 ± 20.84	123.4 ± 3.41^**, #, ^^
Lower 95% CI	197.4	270.1	121.0
Upper 95% CI	236.6	321.9	125.8
ω3	227 ± 7.25	292.6 ± 18.45	120 ± 9.40^**^
Lower 95% CI	218.0	269.7	113.3
Upper 95% CI	236.0	315.5	126.7

^*,#,^^: *p* ≤ 0.05, ^**, # #,^^^: *p* ≤ 0.01, ^****^: *p* ≤ 0.0001. Newborn male rats were not exposed to any treatment during postnatal life.

The body weights of male offspring rats were measured before perfusion. It was found that there was a statistically significant decrease in the body weight of male offspring rats in the EMF group compared to the Cont group (*p* = 0.0001). When the Sham group was compared to the Cont group, a highly significant decrease was seen in this group (*p* = 0.003). There was no significant difference between the EMF-ω3 and EMF groups (*p* > 0.05). However, A significant increase in body weight was found in the offspring rats of the EMF-Mel group compared to those of the EMF group (*p* = 0.036). Also, it was seen that there was a significant decrease in the body weight of the rat in the EMF-ω3 group compared to the Cont group (*p* = 0.0011). The body weight of the rat in the Mel group was higher than that of the rats in the Sham group (*p* = 0.032). In addition, the weights of animals belonging to the Mel and ω3 groups were higher than those of the EMF group (respectively, *p* = 0.0013, *p* = 0.0097). However, there is no significant difference between the Cont, Mel, and ω3 groups (*p* > 0.05) (Table 1; Figure 3).

### 3.2 Antioxidant enzyme levels

**Figure 4:**
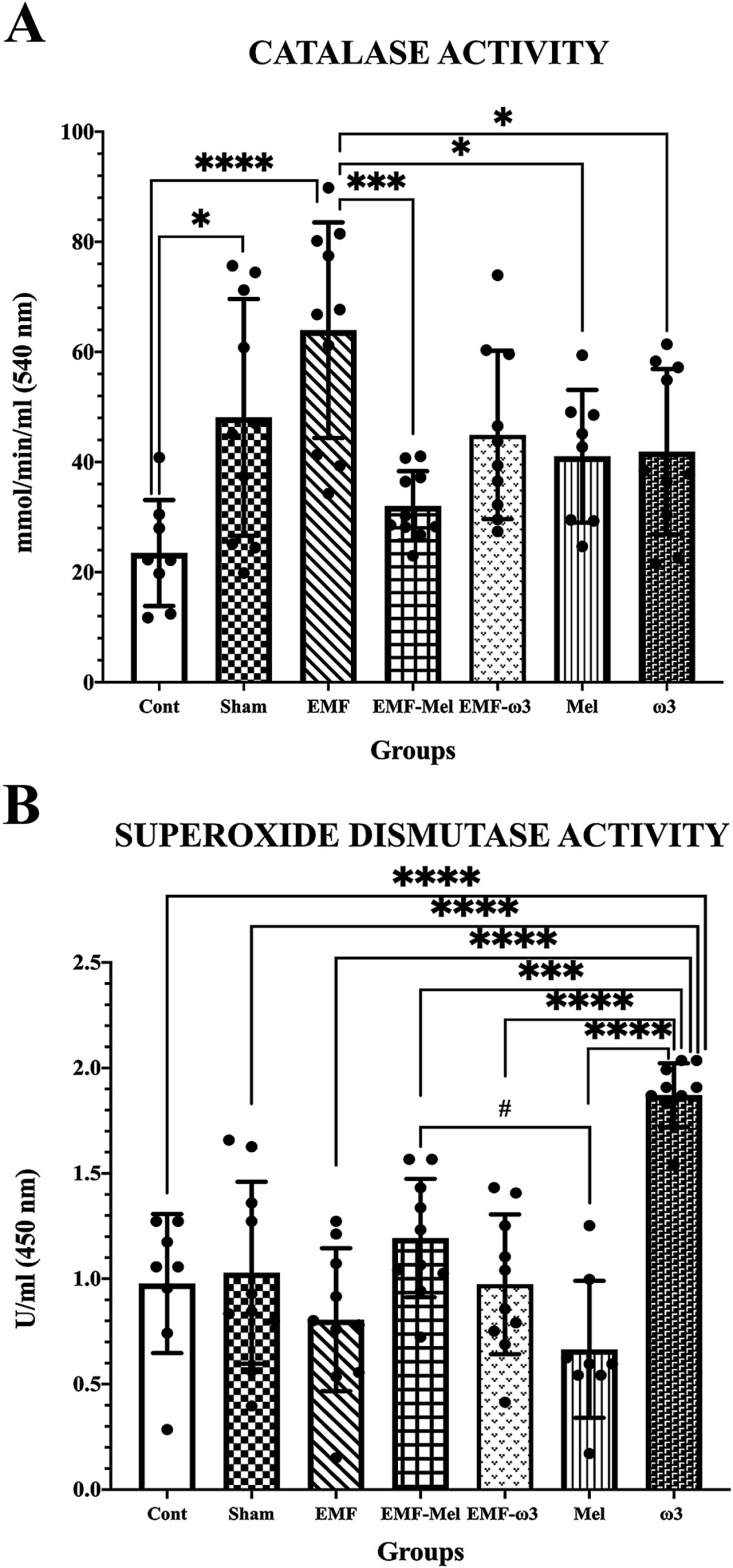
**(A,B)** CAT enzyme activity (nmol/min/ml) and superoxide dismutase enzyme activity (U/ml) values of all groups are shown in the graphs. Differences that are statistically significant at *p* ≤ 0.05 are shown with (*, #), differences that are significant at *p* ≤ 0.001 and *p* ≤ 0.0001 are shown with (respectively ***, ****).

Statistical differences between the groups were compared in terms of the CAT enzyme (F (6, 59) =6.415, *p* ≤ 0.0001, One-way ANOVA). When all groups’ CAT enzyme activity measurements were evaluated, a significant increase was found in the EMF group compared to the control group (*p* = 0.0001). Similarly, a significant increase was observed in the Sham group regarding CAT enzyme activity (nmol/min/ml) compared to the Cont group (*p* = 0.0196). A statistically significant decrease in CAT enzyme activity was observed in the EMF-Mel group compared to the EMF group (*p* = 0.0003). Additionally, a significant increase was found in the EMF group compared to the Mel and ω3 groups (respectively, *p* = 0.038, *p* = 0.032). In addition, statistical differences between the groups were evaluated in terms of the SOD enzyme (F (6, 59) =14.05, *p* ≤ 0.0001, One-way ANOVA). When the superoxide dismutase enzyme activity (U/ml) measurements of all groups were examined, a significant increase was found in the ω3 group compared to the Cont, Sham, EMF, EMF-ω3, and Mel groups (*p* = 0.0001). Also, the ω3 group had a higher superoxide dismutase (SOD) enzyme level than the EMF-Mel group (*p* = 0.0003). A significant increase was observed in the SOD enzyme activity in the EMF-Mel group compared to the Mel group (*p* = 0.017). No statistical difference was found between the other groups (*p* > 0.05) (Table 2; Figure 4).

**Table 2:** Biochemical and behavioral tests of the offspring rats.

Biochemical tests		
Group of animals	CAT enzyme activity (nmol/min/ml)	Superoxide dismutase enzyme activity (U/ml)
Cont (*n* = 8)	23.46 ± 9.62^*, ****^	0.98 ± 0.33^****^
Lower 95% CI	15.42	0.7014
Upper 95% CI	31.50	1.253
Sham (*n* = 10)	48.1 ± 21.49^#^	1.03 ± 0.43^****^
Lower 95% CI	32.73	0.7200
Upper 95% CI	63.48	1.337
EMF (*n* = 10)	63.95 ± 19.58^****, ***, *^	0.81 ± 0.34^****^
Lower 95% CI	49.94	0.5637
Upper 95% CI	77.95	1.048
EMF-Mel (*n* = 10)	32.04 ± 6.3^***^	1.193 ± 0.28^***, #^
Lower 95% CI	27.54	0.9926
Upper 95% CI	36.55	1.394
EMF-ω3 (*n* = 10)	44.92 ± 15.29	0.97 ± 0.33^****^
Lower 95% CI	33.98	0.7370
Upper 95% CI	55.86	1.211
Mel (*n* = 8)	41.04 ± 12.05^*^	0.67 ± 0.33^****, #^
Lower 95% CI	30.97	0.3937
Upper 95% CI	51.12	0.9371
ω3 (*n* = 10)	41.9 ± 15.01^*^	1.871 ± 0.151^****, ***^
Lower 95% CI	31.16	1.763
Upper 95% CI	52.63	1.979

Behavioral tests				
Groups of animals	Elevated plus maze test			
	Open arm entries	Time spent in the open arms	Closed arm entries	Time spent in the closed arms
Cont (*n* = 8)	11.4 ± 4.06^*, ****, **^	224.0 ± 86.29^****^	4.6 ± 2.59^*^	45.2 ± 31.23^##, ****^
Lower 95% CI	8.5	162.3	2.75	22.86
Upper 95% CI	14.30	285.7	6.45	67.54
Sham (*n* = 10)	6.6 ± 2.17^*^	118.4 ± 86.14	7.5 ± 4.45	160.7 ± 59.69^##^
Lower 95% CI	5.05	56.78	4.31	118
Upper 95% CI	8.15	180	10.69	203.4
EMF (*n* = 10)	3.4 ± 2.27^****, ##^	64.9 ± 25.86^****, **, *,^	10.4 ± 4.17^*, **^	238.6 ± 43.66^****, **^
Lower 95% CI	1.78	46.4	7.418	207.4
Upper 95% CI	5.02	83.4	13.38	269.8
EMF-Mel (*n* = 10)	5.6 ± 2.22^**^	173 ± 57.01	4.4 ± 1.71^*^	107.2 ± 59.28
Lower 95% CI	4.01	132.2	3.18	64.79
Upper 95% CI	7.19	213.8	5.63	149.6
EMF-ω3 (*n* = 10)	9.1 ± 4.46^##^	227.3 ± 65.14^**^	5.3 ± 2.3	81.7 ± 57.08^**^
Lower 95% CI	5.911	180.7	3.65	40.87
Upper 95% CI	12.29	273.9	6.95	122.5
Mel (*n* = 8)	7.6 ± 3.98	186.5 ± 62.23^**^	3.9 ± 1.45^**^	88.8 ± 79.47^**^
Lower 95% CI	4.76	142	2.86	31.95
Upper 95% CI	10.45	231	4.94	145.6
ω3 (*n* = 10)	9.3 ± 3.4^##^	208.9 ± 57.38^*^	3.6 ± 1.96^**^	87.7 ± 36.43^**^
Lower 95% CI	6.87	167.9	2.20	61.64
Upper 95% CI	11.73	249.9	4.99	113.8

Groups of animals	Open field test				
	Time spent in the central zone	Time spent in the peripheral zone	Supported rearing	Unsupported rearing	Defecation number
Cont (*n* = 10)	11 ± 6.48	246.9 ± 33.2	10.06 ± 4.65	10.3 ± 3.27^**^	1.3 ± 1.42^*^
Lower 95% CI	6.36	223.2	7.28	7.97	0.29
Upper 95% CI	15.64	270.6	13.92	12.64	2.31
Sham (*n* = 10)	8 ± 9.74	266.1 ± 51.47	5.5 ± 4.65	5.8 ± 4.1	2.1 ± 1.79
Lower 95% CI	1.03	229.3	2.17	2.86	0.82
Upper 95% CI	14.97	302.9	8.83	8.74	3.38
EMF (*n* = 10)	4.10 ± 2.99	285.7 ± 18.8^*^	8.9 ± 6.15	4.3 ± 2.41^**^	3.7 ± 2.16^*^
Lower 95% CI	1.96	272.3	4.5	2.58	2.15
Upper 95% CI	6.25	299.1	13.3	6.02	5.25
EMF-Mel (*n* = 10)	7.4 ± 7.43	266.6 ± 2.87	8.4 ± 2.88	9.4 ± 3.98	2.2 ± 1.14
Lower 95% CI	2.09	245.9	6.34	6.56	1.39
Upper 95% CI	12.71	287.3	10.46	12.25	3.01
EMF-ω3 (*n* = 10)	8.8 ± 5.05	257.5 ± 30.43	10.9 ± 6.01	6.6 ± 2.84	3.9 ± 1.20^*^
Lower 95% CI	5.187	235.7	6.6	4.57	3.04
Upper 95% CI	12.41	279.3	15.2	8.63	4.76
Mel (*n* = 10)	4.3 ± 2.83	239.6 ± 26.4^*^	10 ± 3.68	9.1 ± 5.38	3 ± 2.16
Lower 95% CI	2.28	220.7	7.37	5.25	1.46
Upper 95% CI	6.33	258.5	12.63	12.95	4.55
ω3 (*n* = 10)	7.9 ± 4.15	244.8 ± 23.16^*^	8.9 ± 2.85	10.8 ± 4.19^**^	1.8 ± 1.84
Lower 95% CI	4.93	228.2	6.86	7.81	0.74
Upper 95% CI	10.87	261.4	10.94	13.79	2.86

^*,#^: *p* ≤ 0.05, ^**, # #^: *p* ≤ 0.01, ^***^: *p* ≤ 0.001, ^****^: *p* ≤ 0.0001. Since hemolysis occurred in the blood samples of Cont and Mel groups in biochemical tests, the number of subjects was evaluated as “*n* = 8.” Newborn male rats were not exposed to any treatment during postnatal life.

### 3.3 Open field test

**Figure 5:**
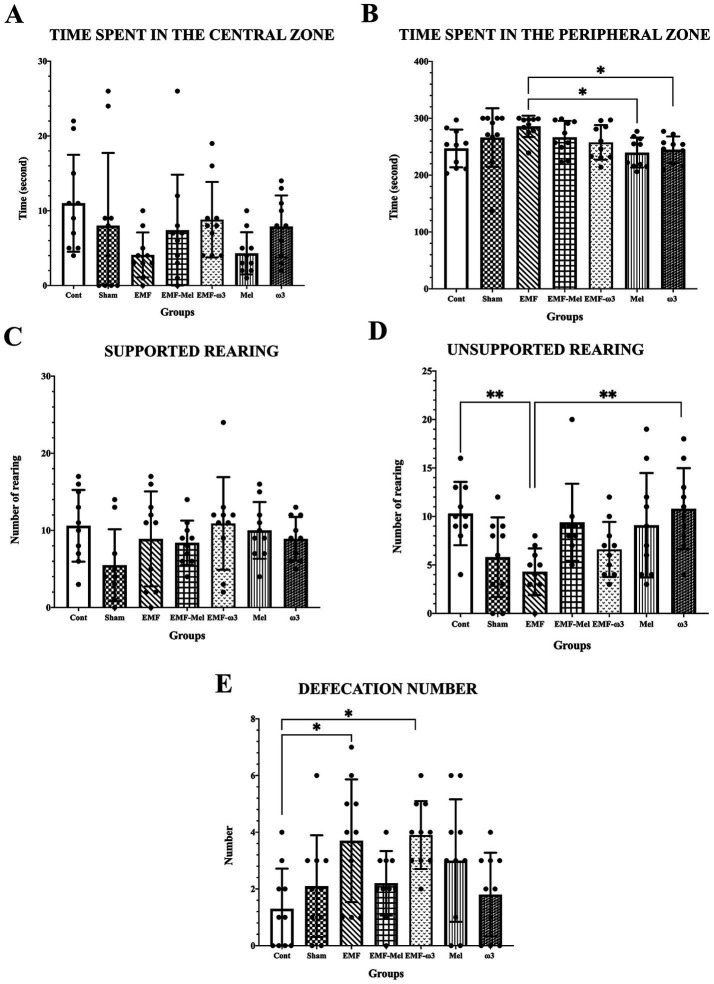
**(A–E)** Graphs for the parameters of the open field test present a statistical comparison of the open field test parameters of all groups: **(A)** the time spent in the center, **(B)** the time spent in the peripheral area, **(C)** supported rearing, **(D)** unsporting rearing, **(E)** the number of defecations. **(A–E)** Data for the groups are expressed as “mean ± standard deviation.” Statistically significant differences at the level of *p* ≤ 0.05 between the groups are shown with (*), and differences at the level of *p* ≤ 0.01 are shown with (**).

Anxiety and locomotor activity were evaluated regarding the time spent in the central zone; it was observed that there was no significant difference between all groups (*p* > 0.05). However, it was found that the time spent in the peripheral zone by the animals in the EMF group was longer than in the Mel and ω3 groups (respectively, *p* = 0.015, *p* = 0.038). On the other hand, when the time spent in the peripheral zone was compared, there was no significant difference between other groups (*p* > 0.05). When the number of unsupported rearing of the offspring rats in the open field test was examined, a significant decrease was found in the EMF group compared to the Cont and ω3 groups (*p* = 0.006, *p* = 0.007). No difference was observed between the other groups (*p* > 0.05). However, when the values of all groups were statistically examined in terms of supported rearing, no difference was found between the groups (F (6, 63) =1.57, *p* = 0.1708, One-way ANOVA). In the open field test, the defecation numbers of the groups were compared statistically (F (6, 63) =3.473, *p* ≤ 0.05, One-way ANOVA). A significant increase was found in the EMF group compared to the Cont group (*p* = 0.0032). Similarly, when the EMF-ω3 group was compared with the Cont group, a significant increase in the number of defecations was found (*p* = 0.015). However, no difference was found between the other groups (*p* > 0.05) (Table 2; Figure 5).

### 3.4 Elevated plus maze test

**Figure 6:**
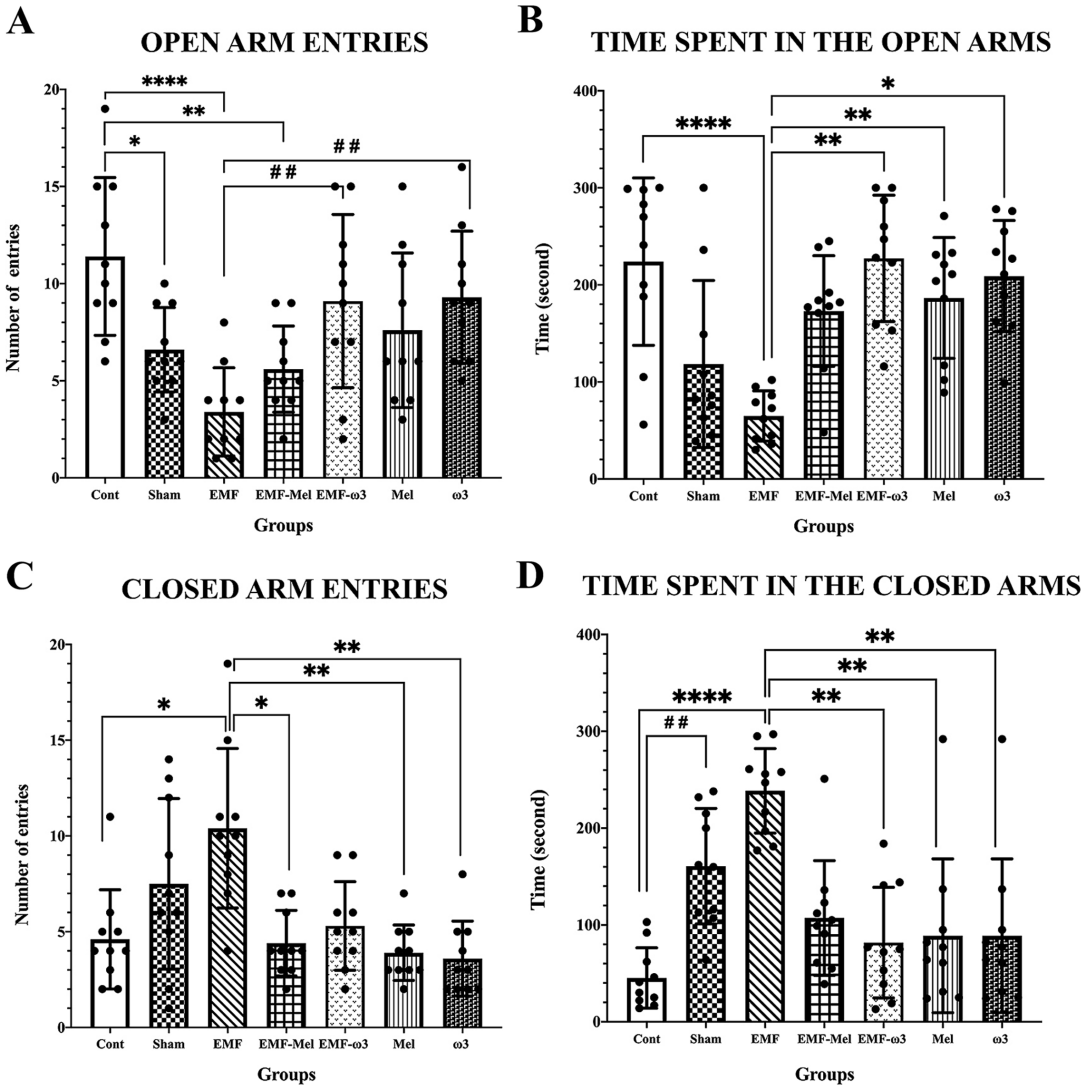
**(A–D)** Graphs for the parameters of the elevated plus maze test is presented: **(A)** number of open area entries, **(B)** duration of stay in the open area, **(C)** number of closed area entries, **(D)** duration of stay in the closed area, and statistical comparison between groups. Values belonging to groups are expressed as “mean ± standard deviation.” Statistically significant differences at the *p* ≤ 0.05 level between groups are shown with (*). Significant differences at the *p* ≤ 0.01 level are shown with (**, # #), and significant differences at the *p* ≤ 0.0001 level are shown with (****).

When elevated plus maze test findings were evaluated, statistical differences between the groups were evaluated in terms of the number of entries into the open area (F (6, 63) =6.269, *p* ≤ 0.0001, One-way ANOVA). A significant decrease was observed in the number of entries into the open area in the Sham group compared to the Cont group (*p* = 0.033). Additionally, a significant decrease was found in the number of entries into the open area of the EMF group compared to the Cont group (*p* < 0.0001). A significant increase was found when the results of the EMF-ω3, ω3 groups were compared to the EMF group (respectively, *p* = 0.006, *p* = 0.004). In addition, a significant decrease in the EMF-Mel group was compared to the Cont group (*p* = 0.005). No statistically significant difference was found between the other groups (*p* > 0.05). When the test findings were evaluated regarding the time spent in the open arms, a significant decrease was found in the EMF group compared to the Cont group (*p* = 0.0005). A significant increase was observed in the EMF-ω3 group compared to the EMF group (*p* = 0.0003). Similarly, a significant increase was observed in the Mel and ω3 groups compared to the EMF group (respectively, *p* = 0.03, *p* = 0.004). No difference was seen between the other groups (*p* > 0.05). When the number of entries into the closed arms was compared, there was a significant increase in the EMF group than in the Cont group (*p* = 0.0038). It was observed that the number of entries into the closed arms in the EMF-Mel group decreased significantly compared to the EMF group (*p* = 0.0038). Additionally, the number of entries to the closed arms in the Mel and ω3 groups was higher than in the EMF group (respectively, *p* = 0.007, *p* = 0.0012). No significant difference was found between the other groups regarding the number of entries into the closed arms (*p* > 0.05). When the elevated plus maze test was evaluated regarding the time spent in the closed arms, it was found that the Sham rats stayed longer than the Cont group rats (*p* = 0.004). A significant increase was observed in the EMF group compared to the Cont group (*p* = 0.0001). When the EMF-ω3, Mel, ω3 groups were compared with the EMF group regarding the time spent in the closed arms, a significant decrease was found (respectively, *p* = 0.002, *p* = 0.002, *p* = 0.015). There was no significant difference in the duration of stay in the closed area between the other groups (*p* > 0.05) (Table 2; Figure 6).

### 3.5 Stereological data

**Figure 7:**
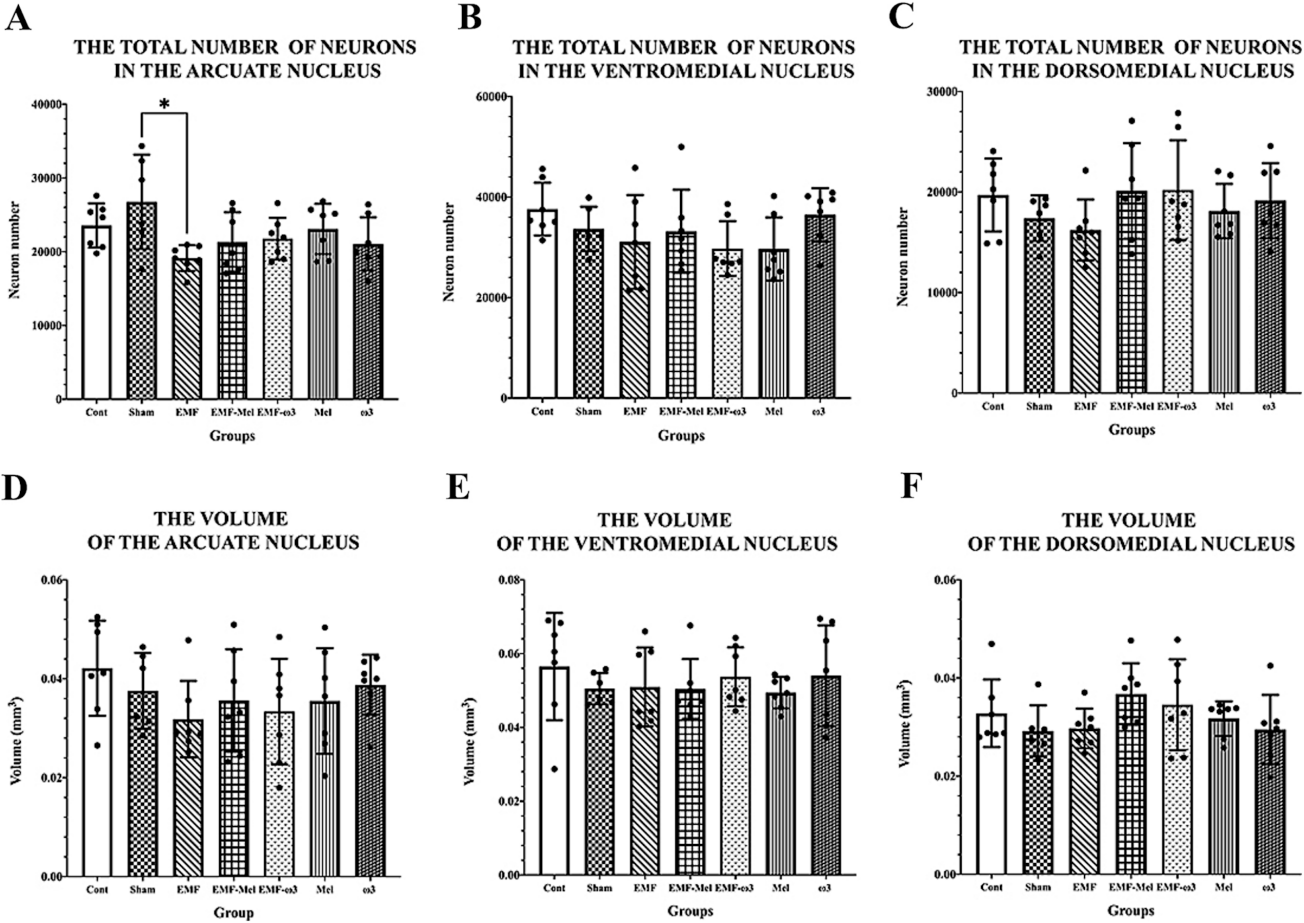
**(A–F)** The graph shows neuronal number and volumetric values of ARN, VMN, and DMN. Neuron numbers and volumes are expressed as mean ± standard deviation. **(A)** A statistically significant difference (*) is seen between the EMF and Sham at the level of *p* ≤ 0.05.

Statistical differences between the neuron number in the ARN of groups were compared (F (6, 41) =2.677, *p* ≤ 0.05, One-way ANOVA). As a result of the statistical evaluation of the total neuron number in the ARN region of the hypothalamus, a highly significant decrease was observed in the total neuron number of the EMF group compared to the Sham group (*p* = 0.012). No significant difference was observed between the other groups regarding neuron number (*p* > 0.05). There was no difference between the groups in terms of the number of neurons in the DMN (F (6, 41) =1.129, *p* = 0.3629, One-way ANOVA). Similarly, there was no difference between the groups in terms of neuron number in the VMN. When the ARN, VMN, and DMN volume values of the hypothalamus were compared statistically, no difference was observed between the groups (*p* > 0.05). Similarly, in terms of the total number of neurons in the VMN and DMN, no significant difference was observed between the groups (*p* > 0.05) (Table 3; Figure 7).

**Table 3:** Stereological results of the offspring rats’ hypothalamic nuclei.

Group of animals	Neuron number			Volume		
	ARN	VMN	DMN	ARN	VMN	DMN
Cont (*n* = 7)	23,562 ± 3,005	37,613 ± 5,235	19,709 ± 3,624	0.042 ± 0.01	0.057 ± 0.015	0.033 ± 0.007
Lower 95% CI	20,782	32,772	16,357	0.033	0.043	0.026
Upper 95% CI	26,341	42,455	23,061	0.051	0.07	0.039
Sham (*n* = 6)	26,764 ± 6412*	33,662 ± 4,428	17,389 ± 2,274	0.038 ± 0.008	0.051 ± 0.004	0.029 ± 0.005
Lower 95% CI	20,035	29,015	15,003	0.03	0.046	0.024
Upper 95% CI	33,493	38,310	19,775	0.046	0.055	0.035
EMF (*n* = 7)	19,155 ± 1754*	31,101 ± 9,315	16,218 ± 3,044	0.03184 ± 0.00773	0.051 ± 0.011	0.03 ± 0.004
Lower 95% CI	17,533	22,486	13,403	0.025	0.041	0.026
Upper 95% CI	20,776	39,716	19,033	0.039	0.061	0.034
EMF-Mel (*n* = 7)	21,311 ± 4,025	33,179 ± 8,265	20,118 ± 4,756	0.03560 ± 0.01039	0.05 ± 0.008	0.037 ± 0.006
Lower 95% CI	17,589	25,535	15,720	0.026	0.043	0.031
Upper 95% CI	25,033	40,823	24,516	0.045	0.058	0.043
EMF-ω3 (*n* = 7)	21,763 ± 2,838	29,760 ± 5,462	20,198 ± 4,953	0.03339 ± 0.01066	0.054 ± 0.008	0.035 ± 0.009
Lower 95% CI	19,138	24,708	15,617	0.024	0.046	0.026
Upper 95% CI	24,388	34,811	24,779	0.043	0.061	0.043
Mel (*n* = 7)	23,081 ± 3,396	29,685 ± 6,281	18,099 ± 2,706	0.036 ± 0.011	0.049 ± 0.004	0.032 ± 0.004
Lower 95% CI	19,939	23,877	15,596	0.026	0.046	0.028
Upper 95% CI	26,222	35,494	20,602	0.045	0.053	0.035
ω3 (*n* = 7)	21,055 ± 3,619	36,497 ± 5,301	19,157 ± 3,736	0.039 ± 0.006	0.054 ± 0.01	0.03 ± 0.007
Lower 95% CI	17,708	31,594	15,702	0.033	0.041	0.023
Upper 95% CI	24,402	41,400	22,612	0.044	0.067	0.036

^*^: *p* ≤ 0.05. In stereological analyses, the number of subjects was calculated by considering the appropriate CV value (≤0.2) (for example, for ARN, the CV values of Cont: 0.12, Sham: 0.22, EMF: 0.08, EMF-Mel: 0.17, EMF-ω3: 0.12, Mel: 0.14, ω3: 0.16). ARN: Arcuate nucleus, VMN: Ventromedial nucleus, DMN: Dorsomedial nucleus. Newborn male rats were not exposed to any treatment during postnatal life.

### 3.6 Light microscopic findings

When the images of the hypothalamic region obtained from the brain tissue of the Cont group were examined, it was observed that the cells and other tissue elements were healthy. Euchromatic nuclei and nucleoli were evident in the neurons. It was observed that the cytoplasm of some cells in the region was darkly stained due to the abundance of granular endoplasmic reticulum. There were many healthy neuroglia cells and blood vessels between the neurons. The nucleus and nucleolus borders of the neurons were evident, and it was observed that the nuclear membranes of the cells in this region had a high degree of invagination. Neurons and neuroglia in the hypothalamic region of the Sham group had normal morphology. Deep invaginations in the nuclei of some neurons were noticeable. Degenerated cells, thought to have lost their function, were also found among the neurons.

**Figure 8:**
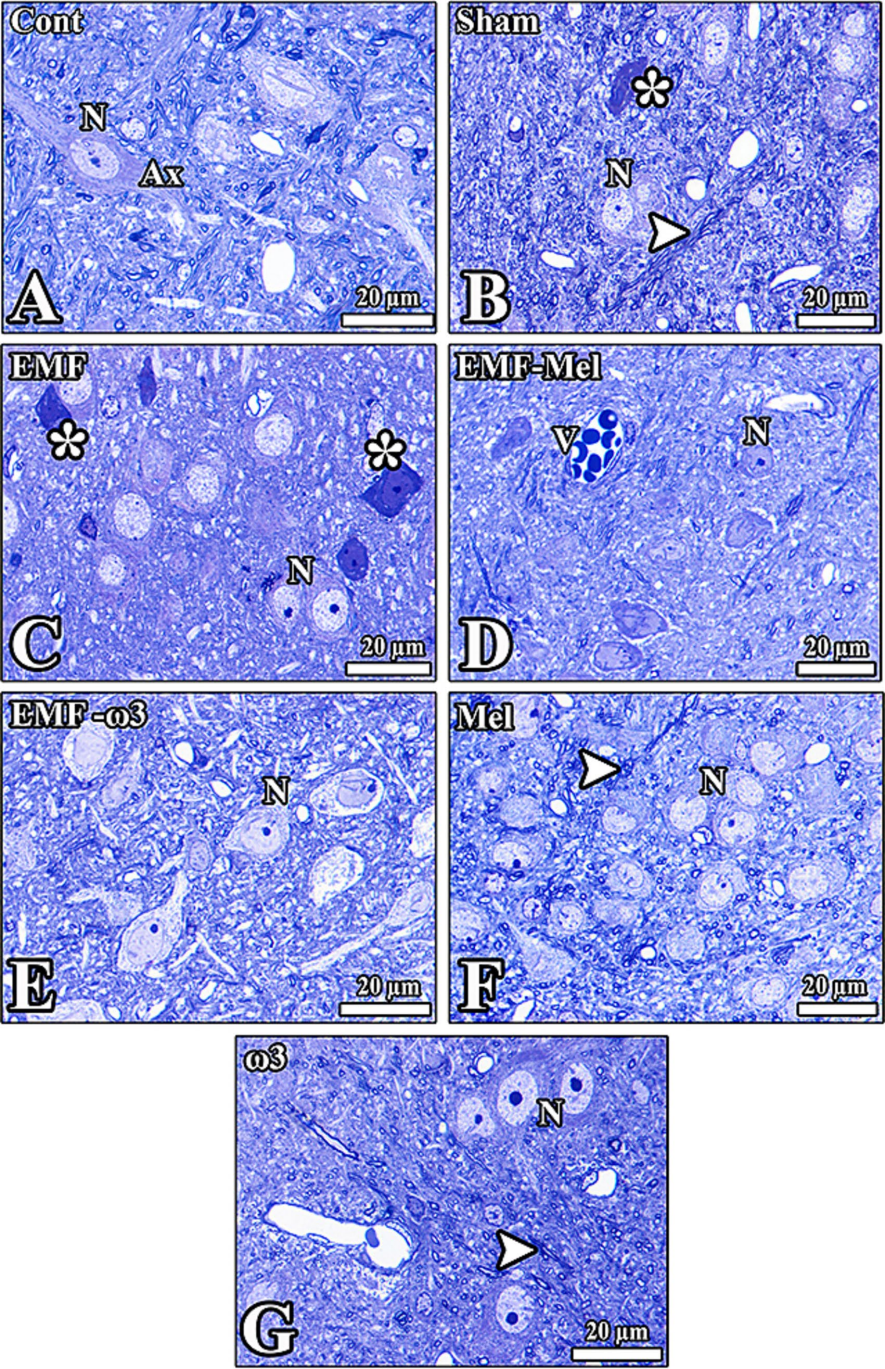
**(A–G)** Histological images from the groups were seen. **(C)** However, healthy neurons (N) and nerve fibers (Ax) in the hypothalamic region of all groups are observed except the EMF-exposed group. The morphology of neurons was seriously impaired (*). V: Vessels in the tissue, degenerated cells (*), and axons (arrowhead) are seen. Semithin sections (0.5 μm) obtained from the resin block were stained with toluidine blue.

Neurons and neuroglia cells of different diameters are observed in the hypothalamic region of the EMF-Mel and EMF-ω3 groups. It was observed that the nucleolus diameters were large and located close to the nuclear membrane in most neurons of the EMF-Mel group. The presence of cells with darkly stained cytoplasm among healthy neurons was striking. Also, in the EMF-ω3 group, there were deep invaginations in the membranes of the nuclei in neurons with a spindle-shaped oval nucleus. When the hypothalamic region of the Mel group was examined, it was observed that the nuclear borders of the neurons with normal morphology were distinct. In addition, it was observed that the neurons of different diameters and the myelinated nerve fibers around them of the ω3 group had normal morphology and that the neuron nuclei were stained euchromatically. Darkly stained glial cells were encountered (Figure 8).

### 3.7 Electron microscopic findings

**Figure 9:**
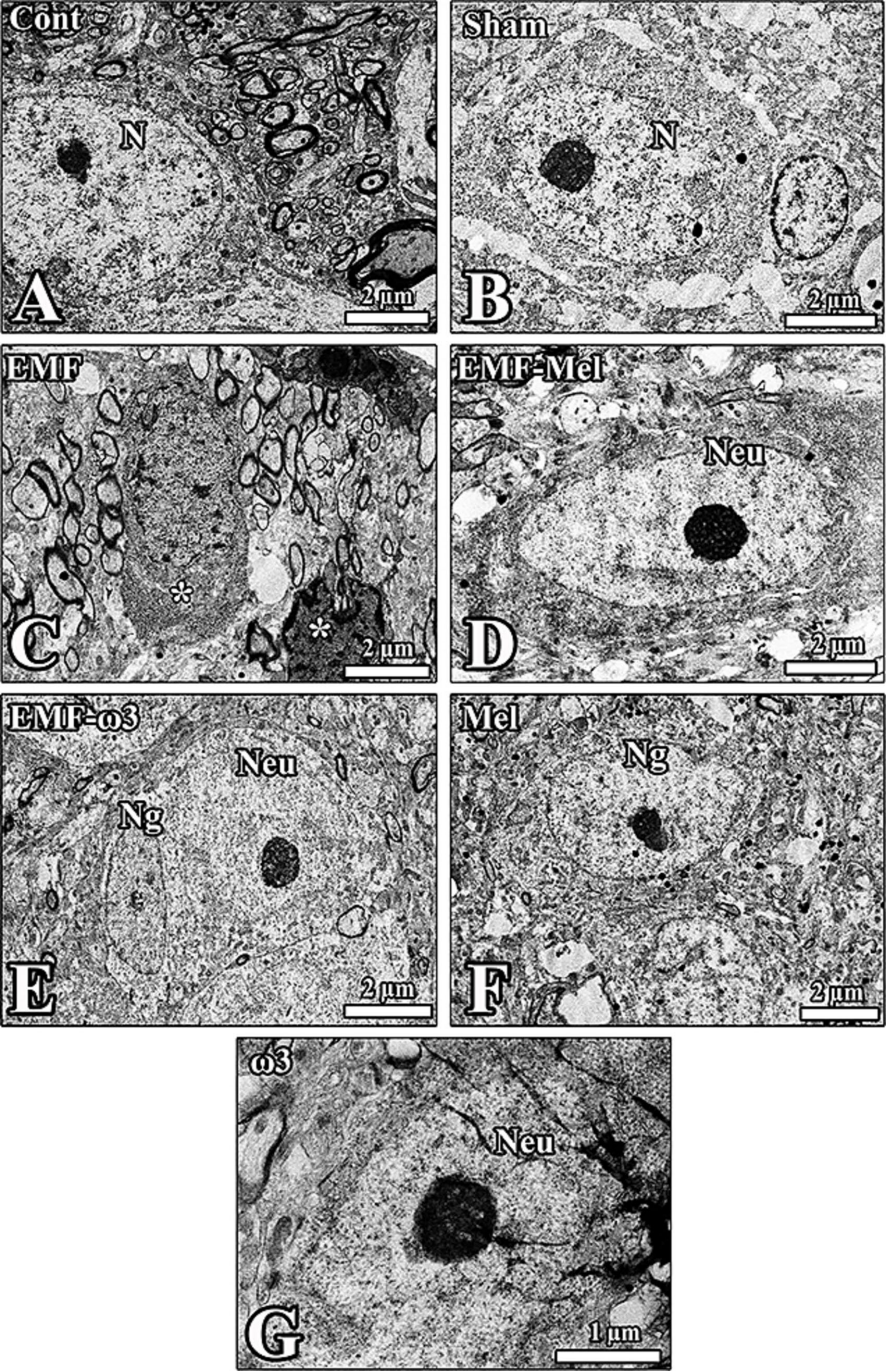
**(A–G)** Electron microscopic images taken from the groups are shown. Neuron (Neu) and neuroglia (Ng) cells in the hypothalamus of all groups are seen. **(C)** The ultrastructural structure of the nucleus (N) of the neurons from all groups is observed, as seen in the degenerated cells in the EMF-exposed groups, which are apparent (*). The impaired shape of neurons and neuroglia was prominent in this group.

It was observed that the neurons and myelinated nerve fibers in the hypothalamic region of the Cont group had a standard structure. Most of the neuron nuclei were centrally located, and the integrity of the organelles in the cell was well preserved. The borders of the cytoplasm and nuclei of the neurons in the hypothalamus of the Sham group were apparent. Darkly stained nucleoli were noticeable in euchromatic nuclei. It was observed that the cells could not maintain their integrity with the surrounding tissue. Deep invaginations were observed in the nuclear membranes. It was observed that most of the neurons and neuroglia cells in the hypothalamic region in the EMF group degenerated. It was noticed that vacuoles formed around the degenerated neuron. Euchromatic stained nuclei and dark-stained nucleoli of neurons in the hypothalamus of the EMF-Mel group were observed. Deep invagination in the nuclei of neurons in the region and well-developed granular endoplasmic reticulum sacs inside the cells were seen. Neurons in the hypothalamic region of the EMF-ω3 group are healthy. It is noticed that the nucleus and nucleolus structures of the neurons and neuroglia cells are healthy. It was noted that the nucleolus diameters in the nucleus were large. It was observed that there were many myelinated nerve fiber sections in the transverse and longitudinal planes around the neurons. The nuclear borders of the neurons of the Mel group were distinct; the nucleolus was close to the nuclear membrane. The large-diameter nucleolus in the euchromatic nucleus was stained darkly. Organelles were distributed homogeneously in the neurons. The neurons and neuroglia cells in the hypothalamus of the ω3 group were healthy. There were also neuroglia cells with normal morphology around the neuron with an euchromatic-stained nucleus. Large invaginations in the nucleus were striking (Figure 9).

### 3.8 Anti-FTO immunolabelling

In the rats of the Cont group, anti-FTO (+) activity was observed in neurons of the ARN region and ependymal cells forming the third ventricle wall. In addition, positively stained areas were seen extensively large in the ARN region, especially in the intercellular medium. In the sham group rats, no anti-FTO (+) staining was observed in the ARN and DMN regions of the hypothalamus. In contrast, anti-FTO (+) staining was observed in ependymal cells located in the wall of the third ventricle. Anti-FTO staining was observed in rats in the EMF group, and low-intensity anti-FTO (+) staining was observed in a small number of neurons in the hypothalamus. While a positive reaction was observed in the cytoplasm in some of the neurons, it was observed in the nucleus in others. In the EMF-Mel group, anti-FTO expression was observed in the intercellular medium around the third ventricle.

**Figure 10:**
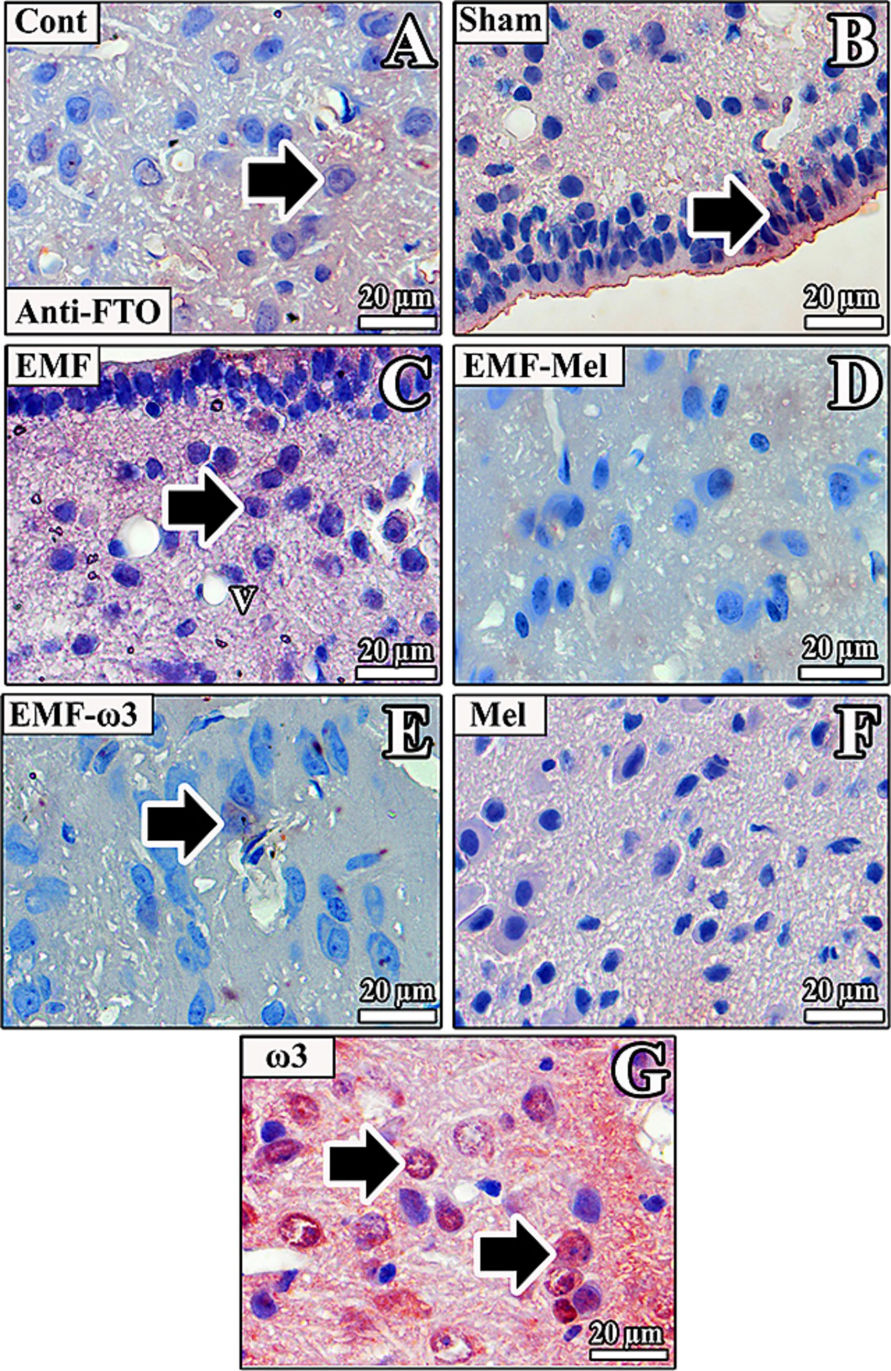
**(A–G)** Neurons in the hypothalamus and ependymal cells of the third ventricle of all groups are observed. Arrows indicate anti-FTO (+) staining. Counterstaining was performed in the sections using Mayer’s hematoxylin.

In contrast, anti-FTO (+) staining was not observed in ependymal cells and neurons. No anti-FTO (+) staining was observed in the neurons of the rats in the EMF + ω3 group. However, low-intensity anti-FTO staining was observed in the intercellular medium. In the Mel group, anti-FTO (+) staining was evident in the ependymal cells’ microvilli in the third ventricle’s wall. In contrast, no positive staining was observed in the neurons in the hypothalamus. No anti-FTO (+) staining was observed in the nuclei of the ependymal cells in the wall of the third ventricle in the hypothalamus of the ω3 group. In contrast, intense positive staining was observed in the intercellular medium. In addition, it was noticed that the neurons in the periventricular hypothalamic nucleus and the intercellular space were positively stained (Figure 10).

### 3.9 Anti-NPY immunolabelling

No anti-NPY (+) staining neurons were observed in the hypothalamus nuclei in the Cont group. It was noticed that there were rare anti-NPY (+) areas in the intercellular medium. In the ARN of the Sham group, anti-NPY (+) staining was observed in the intercellular medium. It was observed that anti-NPY (+) staining was incredibly intense in the intercellular medium. While anti-NPY (+) staining was not commonly observed in the nuclei of neurons and ependymal cells, staining was widespread in the intercellular medium in the subependymal region.

In the EMF group, anti-NPY (+) staining was incredibly intense in the intercellular area. In addition, while the anti-NPY (+) reaction was not observed in the ependymal cells in the wall of the third ventricle, it was especially intense in the intercellular medium in the periventricular hypothalamic nucleus region of the hypothalamus.

In the EMF-Mel group, neurons had low anti-NPY (+) staining intensity. Anti-NPY (+) staining was evident in some neurons in the VMN region of the hypothalamus. In contrast, no positive staining was observed in the ependymal cells in the third ventricle wall.

In the rats in the EMF + ω3 group, high-density anti-NPY (+) staining was observed in the nuclei of neuroglia cells. Positive staining was also observed in the environment around the nerve cells. In addition, a similar anti-NPY (+) staining was observed in the microvilli of the ependymal cells in the third ventricle wall.

**Figure 11:**
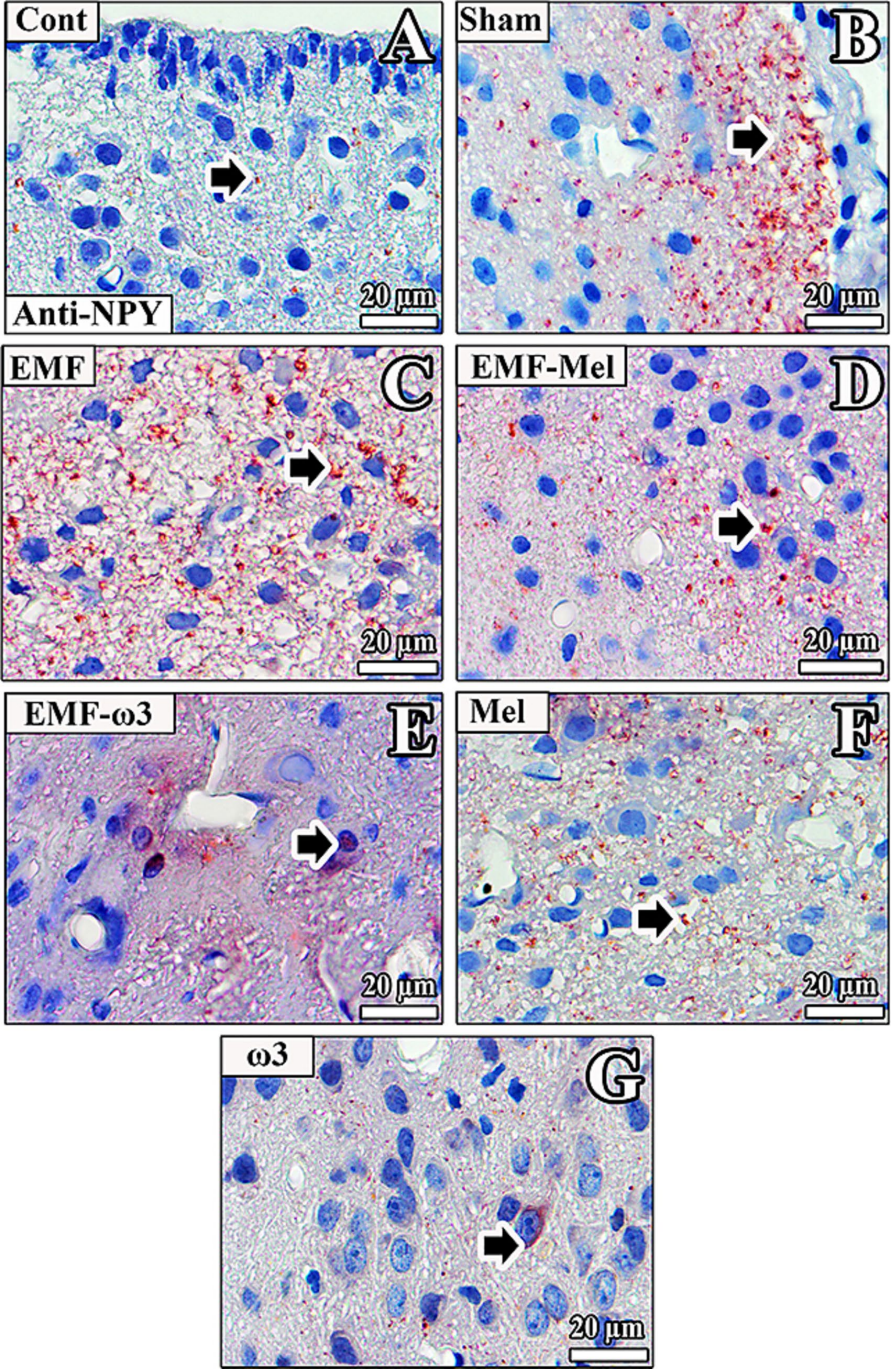
**(A–G)** Neurons in the hypothalamus and ependymal cells of the third ventricle of all groups are observed. Arrows indicate anti-NPY (+) staining. Counterstaining was performed in the sections using Mayer’s hematoxylin.

In the rats of the Mel group, strong anti-NPY (+) staining was observed in interneuron areas. Anti-NPY (+) staining was more evident in the intercellular environment. No staining was observed in the hypothalamus of the ω3 group. However, a low-density anti-NPY (+) staining was observed in the regions close to the third ventricle wall, intercellular environment, and cytoplasm of some neurons in this group (Figure 11).

### 3.10 Anti-Ki67 immunolabelling

In the rats of the Cont group, anti-Ki67 (+) staining was observed to be incredibly intense in the third ventricle wall and regions close to the ventricle wall. Anti-Ki67 (+) staining was observed in the ependymal cell nucleus and the microvilli on the apical surface. In addition, there was anti-Ki67 (+) staining in some neurons in the ARN and VMN. In the Sham group rats, anti-Ki67 (+) staining was not observed in the neurons of the ARN, VMN, and DMN regions. A low-intensity anti-Ki67 (+) staining was observed in the neurons, neuroglia cells, and ependymal cells in the third ventricle wall of the EMF group. In the EMF-Mel group, no anti-Ki67 staining was observed in the intercellular medium. However, low-intensity positive staining was observed in the intercellular medium but not in the cells. Anti-Ki67 (+) staining was not observed in the ependymal cells. Similarly, there was no anti-Ki67 (+) staining in the nuclei of neurons belonging to the EMF-ω3 group, whereas high-density staining was observed in the intercellular space. Positive staining was observed in the nuclei of some ependymal cells in the third ventricle wall and the low-density microvilli of the cells. Anti-Ki67 (+) staining was noticeable in the third ventricle wall of the Mel group. High staining levels were observed, especially in the microvilli of ependymal cells.

**Figure 12:**
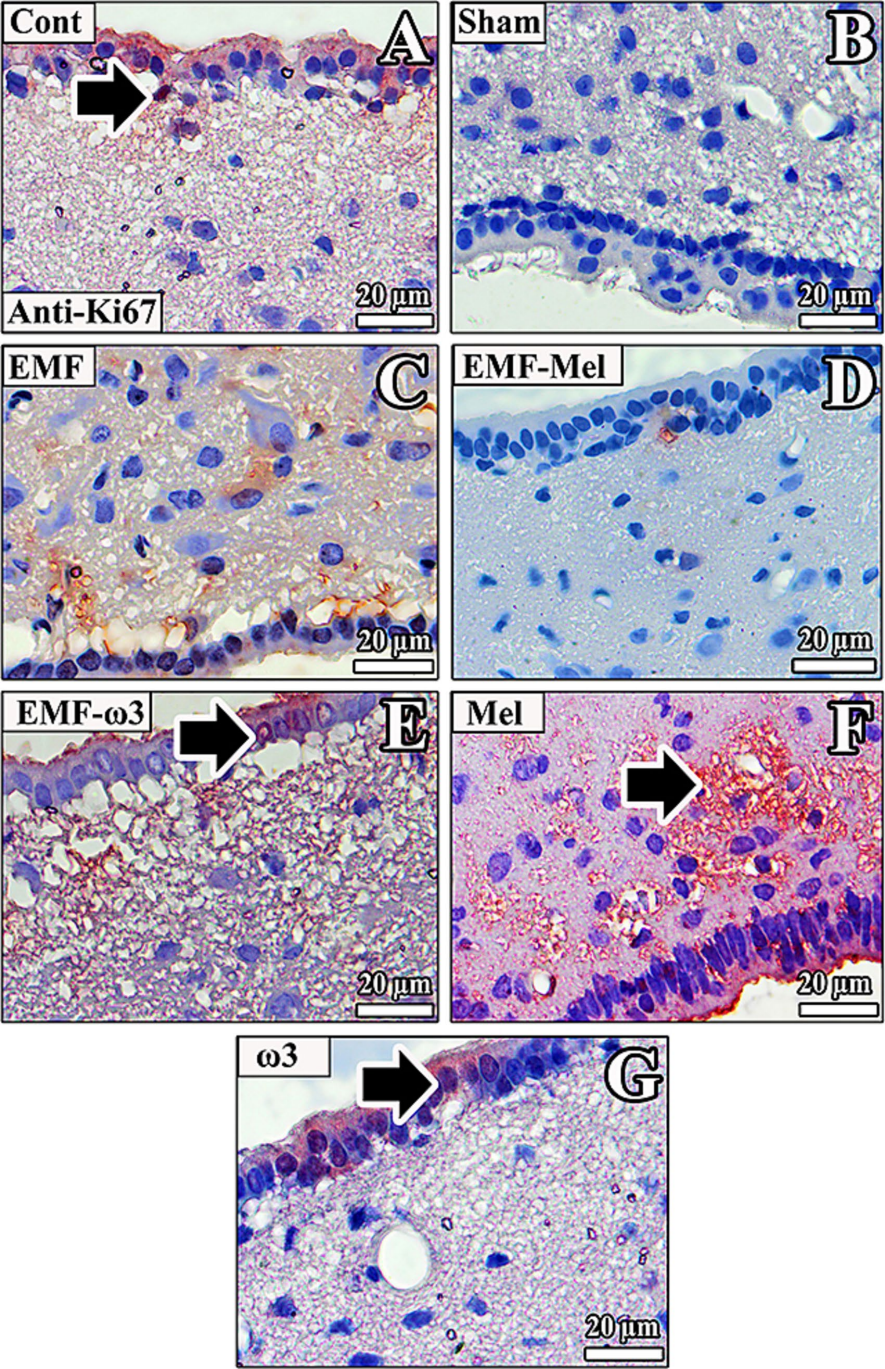
**(A–G)** Neurons in the hypothalamus and ependymal cells of the third ventricle of all groups are observed. Anti-Ki67 (+) staining is indicated by arrows. Counterstaining was performed in the sections using Mayer’s hematoxylin.

On the other hand, no staining was observed in neurons in the ARN, VMN, and DMN regions of the hypothalamus. In the ω3 group, anti-Ki67 (+) staining was observed in the cytoplasm of ependymal cells in the third ventricle wall. At the same time, it was noticed that the nuclei of very few ependymal cells were positively stained (Figure 12).

## 4 Discussion

### 4.1 Expression of the obesity-related genes

In the current study, the hypothalamic effects of EMF emitted from mobile phones were examined in terms of whether they trigger obesity predisposition. The fact that EMF increases the expression of FTO and NPY in the hypothalamus may be evidence that it increases the risk of obesity in infant rats. Considering the open field and elevated plus maze test findings, it can be suggested that EMF may cause stress-induced anxiety. However, more comprehensive studies are needed on this subject. When FTO expression was examined, it was observed that EMF increased expression in the hypothalamus. When EMF was considered a risk factor in hypothalamic obesity predisposition, it was observed that the body weights of offspring in the EMF group decreased compared to the Cont group. Similarly, a decrease in body weight in offspring rats in the EMF group is consistent with that of Dast Amooz et al. (59). They also suggested low brain-derived neurotrophic factor (BDNF) levels were observed after 2,400 MHz EMF exposure in the prenatal period. Long-term epidemiological studies are needed to determine the effect of EMF on hypothalamic obesity.

### 4.2 Association of body weight with obesity-related proteins and genes

When the body weight measurements of mother and offspring rats were evaluated, no difference was observed in the body weight measurements of mother rats belonging to the EMF-ω3 and ω3 groups before pregnancy. However, a decrease in body weight was observed in the EMF-ω3 group compared to the Cont group in the offspring rats. It is seen that Mel and ω3 applied after EMF exposure were not effective in the offspring rats. On the other hand, when only Mel was applied due to the increase in FTO expression, a decrease in the body weight of the offspring rats compared to the Cont group was found, while no difference was observed in the ω3 group compared to the Cont group. It is suggested that ω3 fatty acids do not cause an increase in body weight; it is thought that they may influence body weight due to the calories in the fat (60). The effects of EMF on FTO and NPY expression were observed to be inhibited by Mel and ω3 application. Regarding the effects of mobile phones on FTO, a comprehensive investigation is necessary on the effects of EMF at different frequencies and exposure times on the expression of FTO, the obesity-prone gene.

### 4.3 Assessing the anxiety-related behavioral tests

The increase in the number of defecations in the EMF group in the open plus maze test, the decrease in the number of free standing, as well as the increasing in the number of times in the EMF group entered the dark area and the duration of their stay in the dark area in the elevated plus maze test indicate that prenatal exposure to 900 MHz EMF triggers anxiety in the offspring rats. The increase in the duration of stay in the open area in the Sham group compared to the EMF group provides evidence that the anxiety is not only due to stress. A recent study observed anxiety-like behaviors in rats exposed to ELF-EMF before birth (61).

### 4.4 Stereological estimations and mitotic activity

When the stereological data obtained from the study are evaluated, a decrease in the number of neurons in the ARN after EMF exposure is quite evident compared to the Sham group. Therefore, the EMF effect induced by stress in the behavioral model can be considered. Still, it can be said that the neurodegenerative effect is only due to EMF exposure. The fact that no difference was observed between the Sham and EMF groups in some behavioral test parameters can be associated with the insufficient number of animals in the behavioral tests. With an increase in sample size, the anxiety-triggering effects of EMF can be observed more clearly. The neurodegenerative effect of EMF is more severe in the presence of stress. In addition to the neurodegenerative effect, a low-density anti-Ki67 expression in the Sham, EMF, and EMF-Mel groups compared to the Cont group is also noteworthy. In this context, our study investigated the effect of prenatal EMF exposure in the hypothalamic regions with immunohistochemical markers. Ki67 immunoreactivity was observed prominently in the Cont group, especially in the third ventricle wall and in neurons and neuroglia cells close to the ventricle in the hypothalamus. In the EMF group, Ki67 immunoreactivity was observed prominently in some ependymal cells in the third ventricle wall, compared to the Sham group. It was noticed that Ki67 expression was lower in the EMF group compared to the Cont group. This may be related to the neurodegeneration and mitotic activity-suppressing effect of EMF. The images in the Sham group may be evidence that maternal stress also suppresses mitotic activity in the brain of young rats.

### 4.5 Oxidative stress effects on serum samples

It was observed that 900 MHz EMF exposure increased the level of the CAT enzyme, one of the oxidative stress parameters. When the CAT enzyme level of the EMF exposure group was compared to the control and Sham groups, the level of CAT enzyme increased. In the current study, it was remarkable that the CAT enzyme activity was very high in eliminating increased superoxide radicals in the EMF group. However, the lack of any difference between the SOD enzyme level in the EMF group and the Cont group may be related to the EMF exposure dose and duration of application. It was observed that Mel administered before EMF exposure caused a decrease in CAT enzyme activity. In addition, the absence of any difference between the CAT enzyme activity in the EMF-Mel group and the Cont group shows a positive effect of Mel on CAT enzyme activity. Mel plays an active role in protecting the cell from oxidative stress. However, when our biochemical results were evaluated in terms of superoxide dismutase enzyme activity, it was noticed that there was a difference between the Mel and EMF-Mel groups. It was noticed that there was a low SOD activity in the Mel group. The fact that there was no difference between the Mel and ω3 groups and the Cont group regarding CAT enzyme activity shows that these agents do not cause toxic effects when applied alone. It was observed that the oxidative stress that occurred after EMF exposure did not change with the application of ω3. When the group where ω3 was applied alone was examined, it was seen that there was a significant increase in the SOD enzyme activity in the ω3 group compared to the other groups.

A recent study found that there was a decrease in body weight gain in juvenile rats exposed to RF-EMF in 5G technology. It has been suggested that this effect is insignificant in adult rats exposed to RF-EMF. The decrease in body weight observed in EMF-exposed juvenile rats in our study is consistent with that of Krivova et al. (62). It has been suggested that the RF-EMF exposure in early life has important effects on brain programming and control of eating behavior and body weight (63). The disorder in NPY expression is one of the neurochemical similarities between obesity and depression. NPY-expressing cells were increased after six hours of stress exposure and returned to control levels after 24 h. The increase in the number of NPY-expressing neurons in the ARN was evaluated to be glucocorticoid-induced (64, 65). In addition to evaluating NPY’s excessive hypothalamic expression resulting from EMF at the level of stress response, it is conceivable that it may cause obesity in later life stages due to its orexigenic effects. Overexpression of NPY may lead to obesity. It has previously been suggested that obesity is not caused by hyperphagia, but that the lipogenic effects of NPY play a role in the obesity process (66). In the current study, it can be suggested that EMF triggers anxiety and increases NPY expression, which may trigger a tendency to obesity in male offspring rats. There are not enough studies on the direct effects of EMF on NPY protein. New studies are needed to see the effects of EMF on NPY at the molecular level and elucidate this mechanism.

Accordingly, considering the sensitivity of the fetus to prenatal effects, it is critical to investigate in detail the effects of EMF exposure during the prenatal period on the development of the central nervous system. Another study examining the relationship between prenatal EMF exposure and obesity provided evidence that RF-EMF during embryogenesis may adversely affect molecular mechanisms related to adipogenesis and insulin resistance in zebrafish (67). Future studies are needed to show whether the effects of EMF exposure during the embryonic and prenatal periods may lead to obesity in postnatal life. In an epidemiological study, it was reported that prenatal exposure to high-dose EMF emitted from mobile phones increases the risk of childhood obesity. The growth patterns of children exposed to EMF during pregnancy were investigated (68). Since pregnancy is a highly sensitive period to environmental effects, prenatal EMF exposure can trigger childhood obesity by damaging the development of metabolic and endocrine systems (68, 69). In addition to the hormonal and metabolic factors underlying persistent obesity observed in childhood, it should not be ignored that morphological changes occurring in hypothalamic neurons may increase the risk of predisposition to obesity.

There is insufficient information on the possible effects of EMF on the hypothalamus (23, 70, 71). Any change in neurotransmission observed in the hypothalamic region also significantly affects neurological functions. In this context, 835 MHz EMF exposure affects the energy balance of the hypothalamus and food intake. It has been reported that 835 MHz EMF at 4 W/kg SAR for five hours daily reduces hypothalamic neurotransmission (70). It is suggested that the autophagy response in the cell after 12 weeks of EMF exposure shows a neuroprotective function by preventing neuronal cell death. It is stated that autophagy and apoptosis can coincide in the cell due to their different sensitivity thresholds (71). In our study, an increase in the number of darkly stained neurons in the EMF group was observed in the hypothalamic region. It is thought that these neurons are involved in the apoptosis process. Compared to the Sham group, a higher number of degenerated neurons in the EMF group may indicate the adverse effects of EMF exposure in the hypothalamus that was exposed to EMF during the prenatal period. Apoptosis in hypothalamic neurons plays an essential role in developing and maintaining obesity. It is known that neurogenesis has a restorative effect in the hypothalamic region as well as in the hippocampal region (72). In this context, we think that ω3 protects against the apoptotic effects of EMF exposure. It was observed that the protective effect of ω3 was more pronounced in hippocampal neurons than in Mel’s neuroprotective effects.

The apoptotic effect in the hypothalamus after EMF exposure can lead to obesity. In this context, the neuroprotective effect of ω3 in the hypothalamus can be considered in terms of preventing obesity. On the other hand, observing hypothalamic Ki67 immunoreactivity, especially in the ependymal cells in the hypothalamus and the subependymal region, suggests the neurogenesis-inducing effect of ω3. In a previous study, the subependymal region has been reported to be a niche area in neural stem cell proliferation (73). It can be suggested that ω3 can be used as a therapeutic agent against hypothalamic obesity. Similarly, when ω3 was applied alone, it was observed to inhibit NPY expression in the hypothalamus.

In addition to neurogenesis observed in the subventricular and subgranular regions of the dentate gyrus in the brain, the subependymal niche in the hypothalamus is considered a neurogenic region (73, 74). Additionally, the stress caused by EMF exposure may inhibit mitotic activity. Mel application induces the proliferation of neural stem cells, stimulating the differentiation of stem cells into mature neurons by providing neural differentiation (75). An intense Ki67 immunoreactivity in ependymal cells in the third ventricle wall of the hypothalamus in the Mel group may be due to neurogenesis activity in the subependymal region. However, intense mitotic activity was not observed in the hypothalamic regions in the EMF-Mel group.

## 5 Conclusion

In conclusion, in addition to the effects at the protein level, morphological changes in the cell’s ultrastructure should also be considered for the predisposition to obesity. Our study found neurodegenerative effects in hypothalamic nuclei following EMF exposure. When stereological data were considered, it was observed that the number of neurons decreased because of EMF exposure, especially in the ARN of the hypothalamus. The degenerative effect of EMF exposure was observed in the ARN.

There is no study whose results are supported by qualitative and quantitative evaluations on the effects of EMF exposure during the prenatal period. Studies are needed to examine protein values at the tissue level after EMF exposure and explain the underlying mechanisms. Prenatal exposure to EMF may create a risk of predisposition to obesity. More studies are needed on the protective and healing effects of Mel and ω3 on the prenatally EMF-exposed subjects.

## Data Availability

The original contributions presented in the study are included in the article/supplementary material, further inquiries can be directed to the corresponding author.
